# Lymph Node Ratio in Head and Neck Cancer with Submental Flap Reconstruction

**DOI:** 10.3390/biomedicines10112923

**Published:** 2022-11-14

**Authors:** Hidenori Suzuki, Shintaro Beppu, Daisuke Nishikawa, Hoshino Terada, Michi Sawabe, Nobuhiro Hanai

**Affiliations:** Department of Head and Neck Surgery, Aichi Cancer Center Hospital, Nagoya 464-8681, Aichi, Japan

**Keywords:** lymph node ratio, squamous cell carcinoma, head and neck, submental artery flap, survival

## Abstract

This study aimed to investigate the relationship between the lymph node ratio (LNR) and survival results of patients with head and neck squamous cell carcinoma (HNSCC) reconstructed by a submental artery flap (SMAF) to limit tumor size. This study retrospectively recruited 49 patients with HNSCC who underwent both primary resection and neck dissection with SMAF reconstruction. The LNR was the ratio of the number of metastatic lymph nodes to the sum number of examined lymph nodes. A LNR of 0.04 was the best cut-off value for HNSCC-specific death on receiver operating curve analysis. Patients with LNRs > 0.04 were univariately related to cancer-specific, disease-free, distant metastasis-free, and locoregional recurrence-free survival than those with LNRs ≤ 0.04 by log-rank test. In a Cox’s proportional hazards model with hazard ratio (HR) and 95% confidence interval (CI) adjusting for pathological stage, extranodal extension and or surgical margins, the LNR (>0.04/≤0.04) predicted multivariate shorter cancer-specific (HR = 9.24, 95% CI = 1.49–176), disease-free (HR = 3.44, 95% CI = 1.23–10.3), and distant metastasis-free (HR = 9.76, 95% CI = 1.57–187) survival. In conclusion, LNR for patients of HNSCC with SMAF reconstruction for limited tumor size was a prognostic factor for survival outcomes.

## 1. Background

Pathological metastasis of lymph nodes was recognized as a prognostic factor of survival outcomes in various types of carcinoma [1,2]. The lymph node ratio (LNR), which was defined as the ratio of the number of lymph node metastasis to the number of resected lymph nodes, was a pathologically simple continuous variable with the reflection of surgery, sampling, and staging [3]. The LNR, regardless of several patterns for neck dissection, has been widely adopted as a survival predictor for head and neck squamous cell carcinoma (HNSCC) [4]. Moreover, the LNR in our institution also predicted survival results for 46 cases of hypopharyngeal squamous cell carcinoma (SCC) from 2000 to 2015 [3] and 35 cases of oral SCC from 2008 to 2013 [5].

The submental artery flap (SMAF) is a regional flap, which was firstly described in 1993 [6], was globally developed as a useful flap for medium-sized surgical defects for HNSCC from retrospective and prospective studies [7,8]. The SMAF with both less invasive procedures and good oncologic results was evaluated as a game-changer reconstruction without microvascular anastomosis in comparison to free-flap reconstruction [9]. The subsite of head and neck cancer is heterogenous. To date, the prognostic value of LNR should be assessed for individuals with SMAF for HNSCC.

Therefore, this research purposed to investigate the association between LNR and survival outcomes for patients of HNSCC treated by surgery with SMAF reconstruction.

## 2. Methods

This retrospective observational study at the Department of Head and Neck Surgery in our hospital, following the Declaration of Helsinki, was carried out and approved by our hospital review board (receipt number of 2019-1-427). Of the 53 patients with HNSCC who were newly diagnosed without distant metastasis and underwent tumor resection with SMAF reconstruction from March 2009 to March 2020, four patients who received no neck dissection were excluded. Therefore, 49 patients who had pathological diagnoses of lymph nodes for interventions and examinations with informed consent were recruited. The treatment strategy using SMAF in this cohort mainly applied to small or intermediate defects in patients with advanced age or exhibition of comorbidity.

## 3. Submental Artery Flap

**Figure 1:**
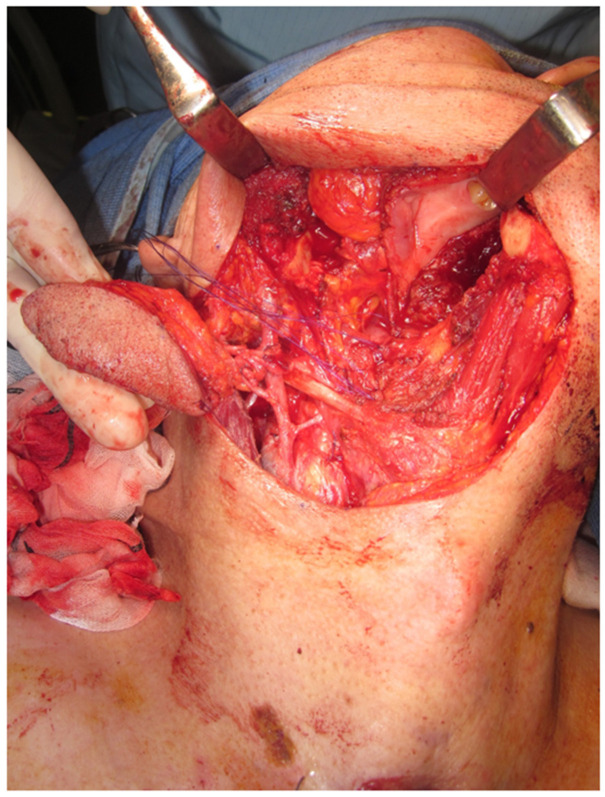
Submental artery flap in right side.

The SMAF was made by head and neck surgeons and is similar to the supraclavicular artery flap as previously described [10]. The SMAF was designed by a pinch test at the submental area of the primary tumor side. The SMAF was designed with both the anterior belly of the digastric muscle and the partial mylohyoid muscle elevated by preserving the submental artery, submental vein, and facial marginal nerve. Primary tumor resection as well as neck dissection were performed by preserving the elevated SMAF. The defect for primary tumor resection was carefully covered without tension by the SMAF. Figure 1 shows a representative image for elevated SMAF.

## 4. Clinicopathological Parameters

The median ± standard deviation of age was 67 ± 12.0 years old. Clinical Tumor, Node, Metastasis (TNM) staging was diagnosed by appropriate images as previously reported [3]. Bilateral neck dissection was recommended for clinical metastasis of bilateral metastases of lymph node or floor of the mouth as primary tumor subsites. The primary sites in the head and neck were oral cavity (*n* = 37), oropharynx (*n* = 8), and hypopharynx (*n* = 4). There were two patients of positive status and six patients of unknown status for human papilloma virus in oropharyngeal cancer. Each pathological restaging of SCC in the primary site was conducted following the seventh edition of the International Union Against Cancer [11]. Experienced pathologists determined the pathological TNM diagnosis with both surgical margins for resected primary tumor and extranodal extension for the metastatic lymph node. The calculation for the LNR was the number of involved lymph nodes relative to the total number of dissected lymph nodes [3]. The median ± standard deviation of primary tumor sizes was 23 ± 13.3 mm based on maximum size from pathological and surgical reports. The main regimen of preoperative chemotherapy was 5-fluorouracil and cisplatin. The main purpose for using induction chemotherapy by 5-fluorouracil and cisplatin was for maximum organ preservation as previously described [12]. Postoperative treatment was recommended by the presence of multiple metastases of lymph node, positive surgical margins, and extranodal extension from pathological reports. Locoregional recurrence for follow-up was performed by salvage treatment as possible.

## 5. Statistical Analysis

The Kaplan–Meier method was applied to calculate survival duration from SMAF reconstruction to a target outcome or last date of contact. The target outcome for each survival type was death from HNSCC to cancer-specific survival (CSS), recurrence or metastasis to disease-free survival (DFS), local or regional recurrence to locoregional recurrence-free survival (LRRFS), distant metastasis to distant metastasis-free survival (DMFS), and death to overall survival (OS). Versatile cut-off values for the LNR were assessed for HNSCC specific death by a receiver operating curve (ROC) analysis with the area under the curve (AUC), as performed by other groups previously [13]. All patients were distinguished into two categories (those with LNR of ≤0.04 vs. >0.04). The comparisons between the two categories in clinicopathological parameters (age, sex, pathological T and N classification, pathological stage, primary tumor size, primary site, positive surgical margin, extranodal extension, type of neck dissection, postoperative treatment, preoperative chemotherapy, smoking history, and extranodal extension and or positive surgical margin) or survival results were assessed by Fisher’s exact test or the log-rank test, respectively. A Cox proportional hazards models with hazard ratio (HR) and 95% confidence interval (95% CI) was used to evaluate multivariate analyses of CSS, DFS, DMFS, and LRRFS. The interaction between LNR and extranodal extension was assessed by the Mann–Whitney U test. Statistical analyses were executed using the JMP software (version 9, SAS: Cary, NC, USA), and *p*-values < 0.05 were considered significant.

## 6. Results

The median number ± standard deviation of positive lymph nodes and the sum of harvested lymph nodes was 1 ± 2.55 and 29 ± 13.8, respectively. The mean and median ± standard deviation of LNR was 0.03, and 0 ± 0.08, respectively. Table 1 presents the associations between LNR and clinicopathological parameters.

The median follow-up ± standard deviation at last contact in the study was 5.04 ± 2.45 years for whole cases, 5.13 ± 2.13 years for the 35 survivors, 2.74 ± 2.52 years for the 14 cases who died, and 1.75 ± 1.32 years for the 10 cases who died from HNSCC. Local recurrence was observed in 10 patients, regional recurrence in 14, and distant metastasis in 9. The 5-year rates of CSS, DFS, LRRFS, DMFS, and OS were 78.0%, 60.5%, 65.7%, 80.6%, and 75.8%, respectively.

**Figure 2:**
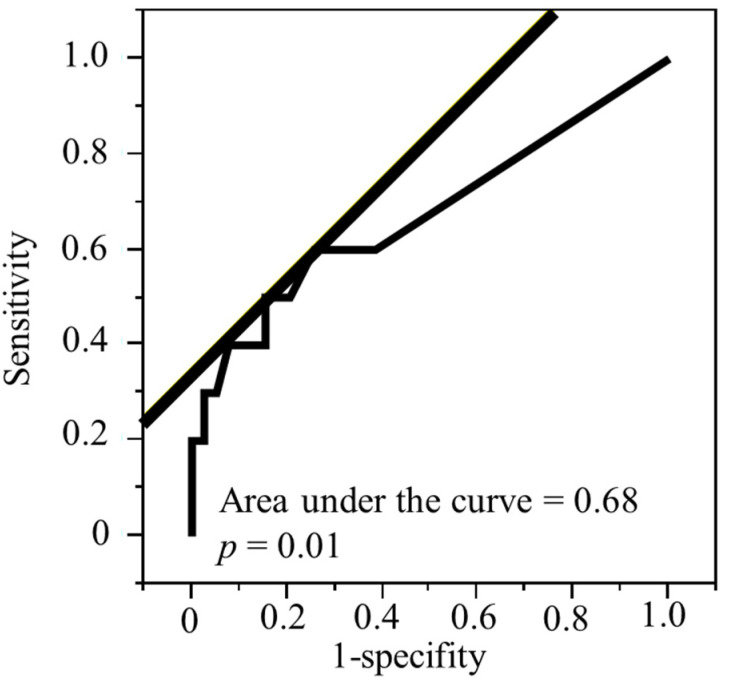
Receiver operating curves for 49 patients with head and neck squamous cell carcinoma.

Figure 2 shows the ROC, the AUC of the ROC for death from HNSCC, 1-specificity, and sensitivity. The optimal cut-off values for LNR to find HNSCC specific death was 0.04 (AUC = 0.68, *p* = 0.01). The sensitivity and specificity in this ROC model were 0.5 and 0.15, respectively. Patients were separated into two categories based on the LNR of 0.04.

**Figure 3:**
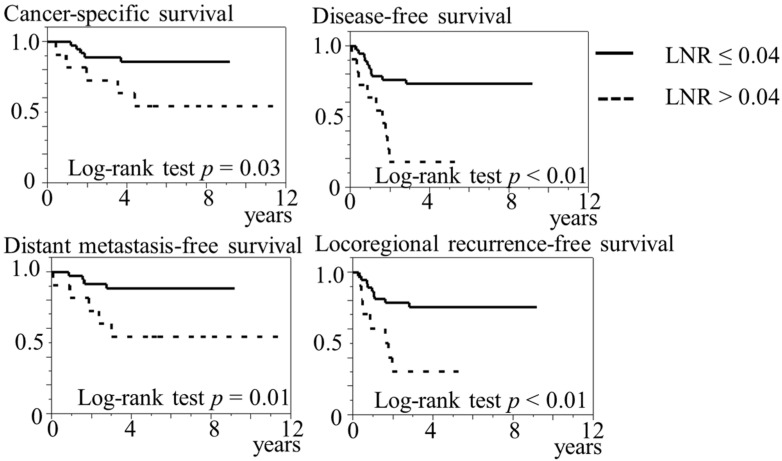
Kaplan–Meier curves in 49 patients were divided into two groups of lymph node ratios. LNR = lymph node ratio.

Figure 3 presents the Kaplan–Meier curves of the two categories for LNR. The log-rank test significantly showed that the group with LNR of >0.04 (*n* = 11) was related to shorter CSS (*p* = 0.03), DFS (*p* < 0.01), DMFS (*p* = 0.01), and LRRFS (*p* < 0.01) in comparison to the group with LNR of ≤0.04 (*n* = 38). Conversely, no significant relationship was found in OS between the two groups for LNR (*p* = 0.32).

Table 2 shows the relationship between clinicopathological parameters and the two categories. Pathological N1-N2 (*p* < 0.01), pathological stage III-IVA (*p* < 0.01), and the presence of postoperative treatment (*p* < 0.01) were more frequently in LNR of >0.04 compared with LNR of ≤0.04.

Table 3 presents the multivariate analyses. The LNR (>0.04/≤0.04) were significantly shorter CSS (*p* = 0.02, HR = 9.24, 95% CI = 1.23–176), DFS (*p* = 0.02, HR = 3.44, 95% CI: 1.23–10.3), and DMFS (*p* = 0.01, HR: 9.76, 95% CI: 1.57–187). No significant associations were found between LNR (>0.04/≤0.04) and LRRFS. Neither pathological Stage (III-IVA/I-II) nor extranodal extension and or positive surgical margin (presence/absence) were associated with survival results.

The LNR of patients with the presence of extranodal extension was a significantly higher value than those with the absence of extranodal extension (*p* < 0.01).

## 7. Discussion

The present study demonstrated using both univariate and multivariate survival analyses, adjusted with pathological stage and extranodal extension and or positive surgical margin, that a significant association existed between higher LNR and shorter CSS, DFS, and DMFS in patients with HNSCC who underwent by surgery with SMAF reconstruction.

The LNR, as a significant predictor for survival outcomes, was reported in HNSCC by meta-analyses and some individual institutions [3,4,5] and was evaluated for patients with focusing on the surgical procedure [3,4,5]. For example, the LNR in 79 patients after primary total laryngopharyngectomy was an independent predictor for OS, CSS, and DFS in univariate and multivariate analyses [13]. Furthermore, the LNR for 327 patients following minimally invasive esophagectomy also predicted OS [14]. Patients with focusing SMAF reconstruction in HNSCC revealed a significant relationship between survival outcomes and LNR, and are similar to previous results [13,14].

Several prognostic factors following SMAF reconstruction in HNSCC were investigated [9,15,16]. Among patients with both tumor resection and SMAF reconstruction, pathological metastasis of lymph node was related to shorter OS and CSS in 160 cases with T1-2 oral SCC [15], the pathological stage was related to DFS in 1169 cases [9], and N stage and pathological differentiation were related to locoregional recurrence in 229 cases [16]. The relationships between LNR and survival results in patients with SMAF reconstruction were not fully investigated because these studies did not investigate LNR [9,15,16]. Therefore, the present study is thought to contribute to additional research. Although one major problem certainly is dissection of level Ia in patients receiving SMAF reconstruction, we focused on both LNR and SMAF with interesting topics in this special cohort. The SMAF in OSCC is often used for patients with comorbid disorders to avoid long anesthesia or microvascular reconstruction due to safeness. This work combines two interesting topics for head and neck surgeons.

Extranodal extension and or the surgical margin and the pathological stage for multivariate analysis in the present study were selected due to both being possible confounding and comprehensive prognostic factors. As one of approaches derived from the pathophysiological significant relationship between the LNR and survival outcomes in both univariate and multivariate analyses of the present and previous results including meta-analysis [3,4,5], the LNR at operation with SMAF for HNSCC was considered for a pathological indicator for postoperative chemoradiaton or radiation.

The present study contains several limitations. Limited sample size was retrospectively observed by a single institution. Therefore, more utile results with more statistical points should be prospectively assessed by a larger cohort from multi-institutions. Because the tumor staging system used in this study was the International Union Against Cancer of the 7th edition, future study is advised to apply that from the American Joint Committee on Cancer of the 8th edition.

## 8. Conclusions

A high-level LNR in HNSCC was a prognostic factor for survival outcomes after operation with SMAF reconstruction.

## Figures and Tables

**Table 1:** Association between clinicopathologic parameters and LNR.

Parameter		Number	LNR (Mean ± Standard Deviation)
Age	<67 year	25	0.04 ± 0.05
	≥67 year	24	0.04 ± 0.11
Sex	Male	31	0.05 ± 0.10
	Female	18	0.01 ± 0.02
Pathological T classification	T1	16	0.01 ± 0.02
	T2	16	0.06 ± 0.13
	T3	9	0.03 ± 0.02
	T4	8	0.05 ± 0.08
Pathological N classification	N0	27	0
	N1	10	0.05 ± 0.03
	N2a	1	0.03
	N2b	10	0.12 ± 0.15
	N2c	1	0.03
Pathological stage	I	13	0
	II	7	0
	III	10	0.03 ± 0.02
	IVA	19	0.07 ± 0.12
Primary tumor size	<23 mm	21	0.01 ± 0.02
	≥23 mm	28	0.05 ± 0.10
Primary site	Oral	37	0.03 ± 0.09
	Oropharynx	8	0.03 ± 0.01
	Hypopharynx	4	0.08 ± 0.09
Positive surgical margin	Presence	7	0.13 ± 0.19
	Absence	42	0.02 ± 0.03
Extranodal extension	Presence	5	0.14 ± 0.21
	Absence	44	0.02 ± 0.04
Type of neck dissection	Unilateral	45	0.04 ± 0.08
	Bilateral	4	0
Postoperative treatment	Radiation	2	0.27 ± 0.35
	Chemoradiation	4	0.06 ± 0.04
	Chemotherapy	3	0.13 ± 0.09
	Absence	40	0.01 ± 0.02
Preoperative chemotherapy	Presence	6	0.06 ± 0.08
	Absence	43	0.03 ± 0.08
Smoking history	Presence	27	0.03 ± 0.05
	Absence	22	0.04 ± 0.11
Extranodal extension and or	Presence	11	0.10 ± 0.15
positive surgical margin	Absence	38	0.02 ± 0.02

LNR = lymph node ratio.

**Table 2:** Association between clinicopathologic parameters and LNR by Fisher’s exact test.

Parameter		LNR ≤ 0.04	LNR > 0.04	*p*-Value
		(*n* = 38)	(*n* = 11)	
Age	<67 year	19	6	
	≥67 year	19	5	1
Sex	Male	22	9	
	Female	16	2	0.18
Pathological T classification	T1–2	25	7	
	T3–4	13	4	1
Pathological N classification	N0	35	2	
	N1–2	3	9	<0.01
Pathological stage	I–II	20	0	
	III–IVA	18	11	<0.01
Primary tumor size	<23 mm	18	3	
	≥23 mm	20	8	0.31
Primary site	Oral	30	7	
	Pharynx	8	4	0.43
Positive surgical margin	Presence	4	3	
	Absence	34	8	0.18
Extranodal extension	Presence	3	2	
	Absence	35	9	0.31
Type of neck dissection	Unilateral	34	11	
	Bilateral	4	0	0.56
Postoperative treatment	Presence	3	6	
	Absence	35	5	<0.01
Preoperative chemotherapy	Presence	4	2	
	Absence	34	9	0.61
Smoking history	Presence	22	5	
	Absence	16	6	0.51
Extranodal extension and or	Presence	7	4	
positive surgical margin	Absence	31	7	0.24

LNR = lymph node ratio.

**Table 3:** Multivariate survival analyses by Cox’s hazard proportional model.

Parameter	CSS	DFS	DMFS	LRRFS
LNR (>0.04/≤0.04)				
HR	9.24	3.44	9.76	2.77
95% CI	1.49–176	1.23–10.3	1.57–187	0.91–8.65
*p*-value	0.02	0.02	0.01	0.07
Pathological Stage (III–IVA/I–II)				
HR	0.15	1.63	0.26	2.59
95% CI	0.01–1.22	0.43–6.71	0.01–2.29	0.65–12.7
*p*-value	0.08	0.47	0.24	0.18
Extranodal extension and or positive surgical margin (Presence/Absence)				
HR	3.67	1.16	2.00	0.71
95% CI	0.77–19.3	0.39–3.20	0.38–9.75	0.19–2.17
*p*-value	0.10	0.78	0.39	0.56

CSS = cancer-specific survival, DFS = disease-free survival, LRRFS = locoregional recurrence-free survival, DMFS = distant metastasis-free survival, LNR = lymph node ratio, HR = Hazard ratio, CI = confidence interval.

## Data Availability

The datasets of this study are available based on reasonable request of the corresponding author.

## Abbreviations

LNR: lymph node ratio; SCC: squamous cell carcinoma; HNSCC: head and neck squamous cell carcinoma; SMAF: submental artery flap; CSS: cancer-specific survival; DFS: disease-free survival; LRRFS: locoregional recurrence-free survival; DMFS: distant metastasis-free survival; OS: overall survival; ROC: receiver operating curve; AUC: area under the curve; HR: hazard ratio; CI: confidence interval.
